# Prevalence of Osteoporosis in Patients with Type 2 Diabetes Mellitus in the Chinese Mainland: A Systematic Review and Meta-Analysis

**Published:** 2019-07

**Authors:** Yuhao SI, Cenyi WANG, Yang GUO, Guihua XU, Yong MA

**Affiliations:** 1. Laboratory for New Techniques of Restoration & Reconstruction of Orthopedics and Traumatology, The First School of Clinical Medicine, Nanjing University of Chinese Medicine, Nanjing 210023, China; 2. Department of Physical Therapy, Rangos School of Health Sciences, Duquesne University, Pittsburgh, 15282 PA, USA; 3. School of Nursing, Nanjing University of Chinese Medicine, Nanjing 210023, China; 4. Institute of Integrated Traditional Chinese and Western Medicine, Nanjing University of Chinese Medicine, Nanjing 210029, China; 5. Department of Traumatology & Orthopedics, Affiliated Hospital of Nanjing University of Chinese Medicine, Nanjing 210029, China

**Keywords:** Type 2 diabetes mellitus, Osteoporosis, Prevalence, Meta-analysis, China

## Abstract

**Background:**

Some studies have investigated the prevalence of osteoporosis in patients with type 2 diabetes mellitus (T2DM) in China. However, the results were inconsistent. This review was performed to estimate the prevalence of osteoporosis in T2DM patients in the Chinese mainland and to characterize its epidemiology.

**Methods:**

A literature search was conducted utilizing PubMed, Scopus, Web of Science, Cochrane Library, CNKI, Wanfang database from their inception through June 2017. A total of 54 studies evaluating the prevalence rate of osteoporosis in T2DM patients were collected. Prevalence estimates from the individual study were combined utilizing random-effect models in Stata 12.0.

**Results:**

The pooled prevalence rate of osteoporosis in T2DM patients was 37.8%. Notably, osteoporosis was more frequent in females than in males (44.8% vs. 37.0%) and was increased with ageing (over 60: 40.1% vs. below 60: 26.5%). Osteoporosis prevalence was higher in less developed areas than in developed areas (41.0% vs. 32.7%) and almost the same between the southern and northern regions (37.6% vs. 38.2%). The prevalence rate between 2010 and 2017 decreased compared with the period between 2001 and 2009 (42.3% vs. 35.6%). Additionally, the meta-regression suggested that gender and age could significantly influence the estimation of prevalence rates respectively (*P* = 0.011, *P* = 0.022).

**Conclusion:**

Osteoporosis affects more than one-third of T2DM patients in China mainland. Females and older adults more likely require clinical prevention due to a higher prevalence. Further studies are needed to be conducted to incorporate and verify previous results.

## Introduction

With the aging population continuing to grow, there has been a significant rise in the prevalence of Type 2 diabetes mellitus (T2DM). It has been observed in practically all regions of the world, with 415 million people suffering from diabetes worldwide (1). In recent decades, people with diabetes in China have increased on a yearly basis. In 2007, there were about 40 million diabetics in China, and it has been estimated that diabetics in China will reach approximately 42.3 million by 2030 (2–4). By that time, China will replace India as the country with the most diabetes patients worldwide.

Diabetes influences the function of multiple organs in the human body, including the heart, brain, kidney, peripheral nerves, eyes, and feet. Additionally, there can also be more than 100 kinds of complications involved with the disease, and it is currently known as the disease with the most complications (5). Throughout recent years, studies on the correlation between diabetes and osteoporosis have been widely recognized by scholars. Both osteoporosis and diabetes are metabolic diseases, and they have a complicated relationship with each other. It is well recognized that type 1 diabetes mellitus can decrease bone mineral density (BMD) and increase the risk of bone fracture (6), while the correlation between T2DM and osteoporosis remains unclear. Therefore, it is necessary for both physicians and patients to be aware of the prevalence rate of osteoporosis in T2DM patients and to learn the relevant characteristics in the diagnosed populations for the sake of early prevention. At present, no systematic review and meta-analysis regarding the prevalence rate of osteoporosis in T2DM patients in China has been conducted.

The objective of this review was to acquire reference values for the prevalence of osteoporosis in T2DM patients utilizing the meta-analysis method. We also described the characteristics of T2DM patients with osteoporosis based on a large sample size, which is intended to provide evidence for physicians as well as the health supervision department.

## Methods

### Search Strategy

According to the PRISMA (2009) standard, we searched PubMed, Scopus, Web of science, Cochrane Library, CNKI, Wanfang data for relevant studies (updated until June 6, 2017). Subject retrieval words and keywords were as follows: type 2 diabetes mellitus, diabetes, T2DM, osteoporosis, prevalence, and epidemiological investigation. Literature languages are not limited.

### Study Selection

Studies involved in this review met the following criteria: the participants were clinically diagnosed with T2DM in the Chinese Mainland; the study design was a cross-sectional study; the test equipment utilized was the BMD dual-energy X-ray absorptiometry and the parts that were tested consisted of the lumbar or the hip. Notably, the prevalence of osteoporosis in T2DM patients can be directly extracted from literature or indirectly calculated.

### Data Collection and Assessment Processes

The retrieved studies were simultaneously and independently screened by two reviewers based on the previously formulated inclusion criteria. The extraction content mainly consisted of the first author, date of publication, diagnostic criteria, sample size, test instrument, test parts, and prevalence. When research reporting positive detection rate of osteoporosis in more than one part was observed, we chose the positive detection rate of the lumbar vertebra to be the standard. After disqualified and repetitive studies removing, the remaining studies were subjected to full-text reading and re-screening. If the information provided were unclear or disputable, the corresponding author of the study would be contacted for a thorough inquiry. Then, decisions of whether to keep the information or discard it would be made. Throughout the course of this process, all disagreements between reviewers were resolved through discussion.

Included studies were graded in 7 aspects according to the Combie evaluation tool which is as follows (7): the study design was scientific and rigorous; the data collection method was reasonable; the response rate of participants was reported; the total representativeness of samples were favorable; the research objective and methods were reasonable; the power of the test was reported; the statistical method was correct. “Yes”, “no” and “have no idea” were respectively utilized to evaluate each item, which was successively given 1 point, 0 points, and 0.5 points. The total score was 7.0 points (6.0∼7.0 points, 4.0∼5.5 points, and 0∼4.0 points were considered to an A, B and C level of quality respectively).

### Statistical Analysis

The primary analysis in this review was the pooled prevalence of osteoporosis in T2DM patients. Heterogeneity was evaluated using the Cochran Q statistic (*P*< 0.10 was considered to be statistically significant) and was quantified using the *I*^2^ index (where *I*^2^> 30% indicated moderate heterogeneity; *I*^2^> 50% substantial heterogeneity; and *I*^2^> 75% considerable heterogeneity). If the *I*^2^ test indicated a value > 50% which reflected significant heterogeneity, then a random-effect model was carried out. Conversely, a fixed-effect model was implemented. The subgroup analysis was conducted based on sex (male or female), average age (younger or older than 60 years old), region (south or north), economic level (developed or less developed) and date of publication (2001∼2009 or 2010∼2016). Besides, a meta-regression analysis was applied as a means to explore the source of heterogeneity among all the studies. Specifically, the concomitant variable includes the date of publication, the female proportion in subjects, region, sample size, economic level, and average age. Funnel plot with the proportion as the abscissa and the standard error as the ordinate was adopted to reflect the publication bias directly. Begg’s test and Egger’s test were both applied to create a qualitative judgment on publication bias. Additionally, the trim and filling method would be utilized to evaluate the stability of the obtained result if necessary. All the above analyses were conducted using the Stata 12.0 version, and all reported *P* values were two-sided with a statistical significance level of 0.05.

## Results

### Literature Search and Characteristics

**Fig. 1:**
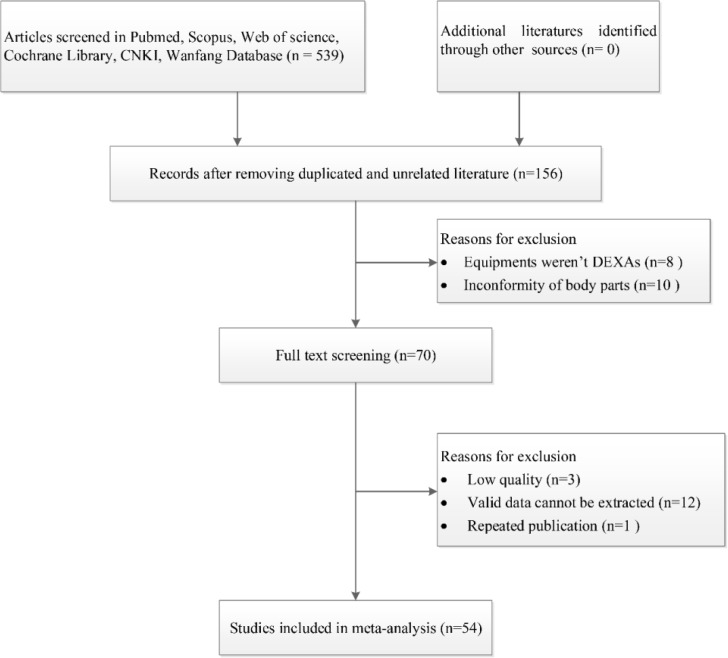
Flow diagram of included/excluded studies

Figure 1 outlines the search strategy and the selection process. Of the 539 relevant studies initially identified, 383 duplicated records were removed, and 68 articles were subsequently excluded after reading titles and abstracts due to their irrelevant clinical question or incorrect publication type.

After the full-text review of 70 studies, we further excluded 16 studies that did not meet the inclusion criteria. Finally, 54 studies with 13462 diabetic patients were included in the meta-analysis (8–61). The majority (79.6%) of the included articles scored greater than 4 points as assessed through the Combie evaluation tool, which indicated high and moderate quality literature. The main characteristics and details of each study are summarized in Table 1.

**Table 1:** Characteristics of included studies

***No.***	***Area***	***Measuring instrument type***	***Measure parts***	***Sample size***	***M/F***	***Age (yr)***	***Quality***
(1)	Hunan	USA Hologic-QDR-4500A	Lumbar, Left Hip	1253	0/1253	59.8±8.61	A
(2)	Liaoning	USA GE Lunar-Prodigy	Lumbar, Femoral Neck	100	18/32	45∼75	B
(3)	Jiangsu	France DMS CHALLENGER	Lumbar, Left Hip	52	24/28	62.05±7.25	B
(4)	Guang dong	NR	Lumbar, Left Hip	165	85/80	60.91±8.91	B
(5)	Zhejiang	USA GE Lunar-Prodigy	Lumbar	72	33/39	55.9±3.3	B
(6)	Jiangxi	France MEDILINK-OSTEOCORE2	Lumbar, Femoral Neck	100	52/48	63.9±6.5	B
(7)	Hebei	France MEDILINK-OSTEOCORE2	Lumbar, Hip	105	59/46	62±11	B
(8)	Hunan	USA Hologic-QDR-4500A	Lumbar, Hip	168	0/168	60.95±12.16	A
(9)	Shanghai	China DEXAUNT2000	Lumbar, Hip	70	29/41	68.26±7.35	B
(10)	Zhejiang	France DMS CHALLENGER	Lumbar, Femoral Neck, Whole Body	100	NR	NR	C
(11)	Sichuan	NR	Lumbar, Hip	230	100/130	NR	C
(12)	Liaoning	Korea OsteoSys-Dexxum T	Lumbar, Femoral Neck	232	64/168	66.1±9.2	B
(13)	Anhui	USA GE Lunar-Prodigy	Lumbar, Hip	118	42/76	51.6±12.5	C
(14)	Anhui	NR	Hip	162	87/75	45∼73	C
(15)	Jiangxi	France DMS CHALLENGER	Lumbar, Left Hip	204	95/109	59.54±10.30	B
(16)	Sichuan	NR	Lumbar, Hip	223	135/88	NR	C
(17)	Tianjin	USA NOLAND-XR-800	Lumbar, Hip	168	80/88	55.6±11.3	B
(18)	Beijing	USA Hologic-QDR-4500A	Lumbar, Hip	306	154/152	69.2±6.8	C
(19)	Beijing	NR	Lumbar, Hip	200	96/104	65.43±8.53	B
(20)	Guizhou	Janpan DAS 600EX	Lumbar	38	38/0	74.0±6.9	C
(21)	Hebei	USA Hologic-QDR-4500A	Lumbar, Hip	158	158/0	65.8±5.7	B
(22)	Beijing	USA GE Lunar-Prodigy	Lumbar, Hip	102	41/61	41.0∼94.4	A
(23)	Heilongjiang	USA GE Lunar-Prodigy	Lumbar, Hip	194	91/103	76.85±4.55	C
(24)	Jilin	USA GE Lunar-Prodigy	Lumbar, Hip	68	68/0	52.85±10.9	B
(25)	Yunnan	NR	Lumbar	137	56/81	59.96±11.73	B
(26)	Sichuan	France MEDILINK-OSTEOCORE2	Lumbar, Left Hip	96	0/51	65.37±7.86	A
(27)	Fujian	USA GE Lunar-Prodigy	Lumbar, Hip	182	97/85	65.11±3.68	B
(28)	Fujian	USA GE Lunar-Prodigy	Lumbar, Hip	85	0/85	NR	C
(29)	Jiangsu	USA GE Lunar-DPX-IQ	Lumbar, Hip	70	70/0	78.5±16.7	B
(30)	Hebei	France MEDILINK-OSTEOCORE3	Lumbar, Hip	150	0/150	62.5±13.6	B
(31)	Henan	USA Hologic-QDR-4500A	Lumbar, Left Hip	355	194/162	NR	C
(32)	Jiangsu	USA Hologic-EXPLORER	Lumbar, Hip	529	268/261	63.07±11.20	B
(33)	Anhui	USA GE Lunar-Prodigy	Lumbar, Left Hip	254	131/123	70.9±9.81	A
(34)	Tianjin	USA GE Lunar-Prodigy	Lumbar, Hip	125	125/0	55.8±11.4	A
(35)	Yunnan	USA GE Lunar-Prodigy	Lumbar, Hip	1218	679/602	63.79±10.33	B
(36)	Hunan	USA Hologic-Delhpi A	Lumbar, Left Hip	3110	0/3110	59.3±10.8	A
(37)	Guangdong	USA NORLAND XR-36	Lumbar, Hip	61	25/36	41∼71	B
(38)	Hunan	USA Hologic-QDR-4500A	Lumbar, Hip	214	0/214	59±8	A
(39)	Henan	USA Hologic-QDR-4500W	Lumbar	52	21/31	69±7	C
(40)	Guangdong	USA GE Lunar-DPX-L	Lumbar, Left Hip	537	203/334	50∼79	B
(41)	Guangdong	USA GE Lunar-DPX-IQ	Lumbar, Hip	103	55/48	61±7	B
(42)	Shanghai	USA GE Lunar-Prodigy	Lumbar, Hip	70	29/41	68.16±7.35	A
(43)	Hebei	USA GE Lunar-DPX-NT	Lumbar, Hip, Distal Radius	50	0/50	60.02±4.13	B
(44)	Hebei	France MEDILINK-OSTEOCORE3	Lumbar, Hip	46	17/29	60.58±9.18	B
(45)	Beijing	USA NORLAND XR-36	Lumbar, Hip	52	52/0	66.0±9.8	B
(46)	Liaoning	France DMS lexxos	Lumbar, Hip	62	0/62	50∼82	A
(47)	Chongqing	China DEXAUNT2000	Lumbar, Hip	78	31/47	71.25±9.46	B
(48)	Hebei	France MEDILINK-OSTEOCORE3	Lumbar, Hip	150	0/150	62.98±12.12	B
(49)	Anhui	USA GE Lunar-Prodigy	Lumbar, Left Hip	302	114/188	50∼70	B
(50)	Hunan	USA Hologic-QDR-4500A	Lumbar, Left Hip	197	0/166	48∼81	B
(51)	Beijing	USA GE Lunar-Prodigy	Lumbar, Hip, Whole Body	104	67/37	59±12	A
(52)	Hebei	France MEDILINK-OSTEOCORE3	Lumbar, Hip, Whole Body	68	0/68	64.79±8.29	B
(53)	Hebei	USA GE Lunar-Prodigy	Lumbar, Hip, Whole Body	106	544/52	59.89±9.60	B
(54)	Hunan	USA Hologic-QDR-4500A	Lumbar, Hip	248	98/150	M: 59.86±11.38 F: 61.25±9.57	A

Abbreviations: M, Male; F, Female; NR, Not Reported

### Pooled Prevalence Rates of Osteoporosis in Diabetic Patients

#### Overall

**Fig. 2:**
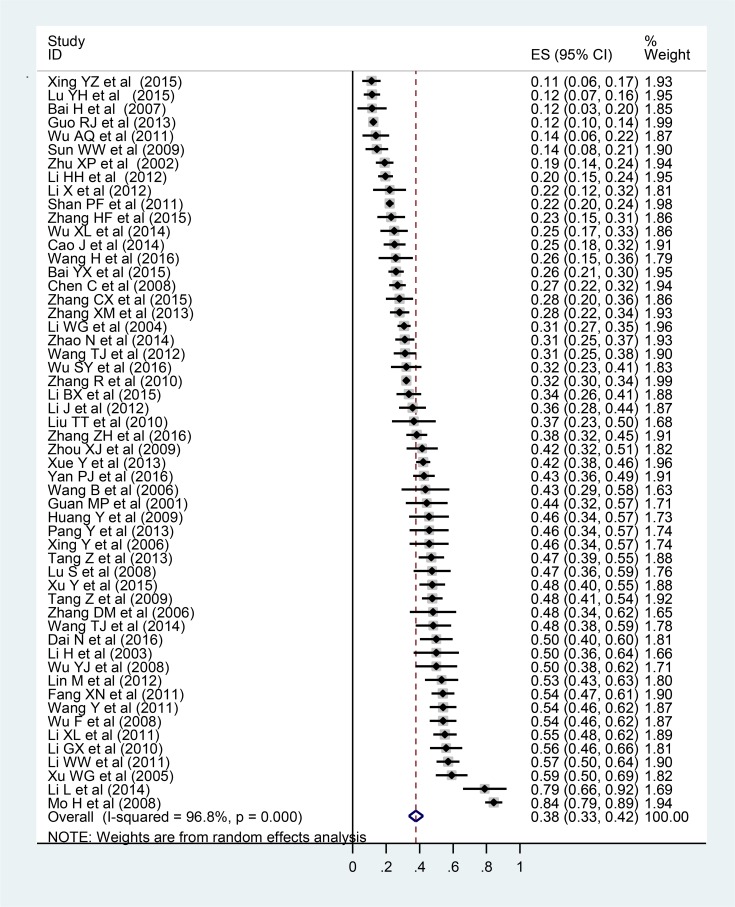
Meta-analysis forest for the prevalence of osteoporosis in type 2 diabetes mellitus patients in Chinese mainland

The pooled prevalence rate of osteoporosis in diabetic patients was 37.8% (95%CI: 33.5%, 42.1%, I^2^=96.8%, *P*<0.001) (Fig. 2). Subgroup analyses were applied based on sex, average age, region, economic level, and publication year in order to explore the source of heterogeneity.

#### Sex

Thirty-one studies and thirty-seven studies reported the prevalence rates of osteoporosis in male and female patients, respectively. The aggregated results indicated that the prevalence rates of osteoporosis in female and male were 44.8% (95%CI: 39.4, 50.2%) and 37.0% (95%CI: 27.5%, 49.8%) respectively (Table 2).

**Table 2:** Prevalence of osteoporosis according to different items

***Category***	***Subgroup***	***NO. of studies***	***N (T2DM)***	***Events (OP)***	***Prevalence, 95%CI (%)***	**P *(Q test)***	**I*^2^(%)***	***Publication bias***	
								***P* (Begg’s)**	***P* (Egger’s)**
Sex	Male	31	3078	716	37.0 [27.5,49.8]	<0.001	90.4	0.905	0.711
	Female	37	8477	2982	44.8 [39.4,50.2]		96.1	0.143	0.001
Average age	<60	12	5679	1649	26.5 [20.8,32.1]	<0.001	94.2	0.373	0.584
	≧60	29	5267	1738	40.1 [33.3,46.9]		96.9	0.511	<0.001
Area	Northern	21	2901	1048	38.2 [31.2,45.1]	<0.001	94.3	0.147	0.007
	Southern	33	10561	3332	37.6 [32.0,43.2]		97.5	0.369	0.008
Economic	Developed	21	3371	1092	32.7 [26.2,39.1]	<0.001	94.6	0.147	0.155
	Less developed	33	10091	3288	41.0 [35.2,46.8]		97.5	0.193	0.001
Publication year	2001∼2009	18	2500	1006	42.3 [32.2,52.4]	<0.001	96.8	0.596	0.553
	2010∼2016	36	10962	3374	35.6 [31.0,40.1]		96.4	0.145	0.002

Abbreviations: T2DM, Type 2 Diabetes Mellitus; OP: Osteoporosis

#### Age

Twelve studies reported that patients average ages were younger than 60 years old, and the other twenty-nine studies reported 60 years old or older. The aggregated results revealed that the prevalence rates of osteoporosis in younger patients and older patients were 26.5% (95%CI: [20.8%, 32.1%]) and 40.1% (95%CI: [33.3%, 46.9%]) (Table 2).

#### Area

We geographically divided China into the southern and the northern regions with the Qinling Mountains-Huaihe River. Twenty-one studies and thirty-three studies were conducted in the southern and northern areas respectively. The aggregated results demonstrated that the prevalence rates of osteoporosis in patients living in the south (38.2%, 95%CI: [31.2%–45.1%]) and north (37.6%, 95%CI: [32.0%–43.2%]) reflected to be nearly the same (Table 2).

#### Economic Level

According to the China’s National Bureau of Statistics, developed regions with a GDP higher than 4 trillion were Beijing, Shanghai, Tianjin, Chongqing, Guangdong, Jiangsu, Shandong, Zhejiang, and Henan. Less developed regions with GDP lower than 4 trillion were Sichuan, Hubei, Hebei, Hunan, Fujian, Anhui, Liaoning, Shanxi, Inner Mongolia, Jiangxi, Guangxi, Heilongjiang, Jilin, Yunnan, Shanxi, Guizhou, Xinjiang, Gansu, Hainan, Ningxia, Qinghai, and Tibet. The pooled results illustrated that the prevalence rates of osteoporosis in patients living in the developed areas and less developed areas were 32.7% (95%CI:26.2%, 39.1%) and 41.0% (95%CI:35.2%, 46.8%) respectively (Table 2).

#### Publication Year

Eighteen studies were published between the 2000 and 2009 period, and 36 studies were published between 2010 and 2016. The merged results demonstrated that the prevalence rates of osteoporosis in diabetic patients from 2010 to 2016 and from 2001 to 2009 were 35.6% (95%CI: [31.0%, 40.1%]) and 42.3% (95%CI: [32.2%, 52.4%]) respectively (Table 2).

#### Regression Analysis and Publication Bias

**Fig. 3:**
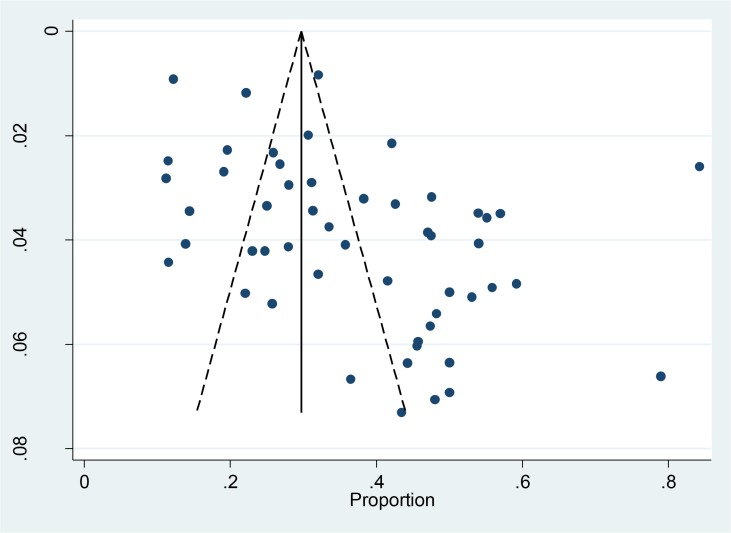
Funnel plot for publication bias

Pre-specified meta-regression designated that publication year, areas, sample size, economic level, and quality scores did not affect the merged prevalence. However, sex (*P*=0.011) and average age (*P*=0.022) in participants remarkably influenced the overall outcome. Significant asymmetry existed in the funnel plot (Fig. 3). The results of the Begg’s test (*P* = 0.058) and the Egger’s test (*P* < 0.001) lacked consistency, which hinted that publication bias existed (Table 3). Therefore, we adopted the trim and fill method to examine the publication bias. The number of trimming and filling study was one after three times of iteration. The *P* value presented no reversal result before and after trimming and filling (*P*<0.001 and *P*<0.001) as it designated that the results were comparatively stable even though the publication bias existed.

**Table 3:** Results of Meta-regression for the prevalence of osteoporosis in type 2 diabetes mellitus

***Covariate***	***Meta-regression coefficient***	***95 % Confidence interval***	**P *value***
Year of publication	−0.008	−0.020 to 0.003	0.164
Female proportion (%)	0.189	0.045 to 0.333	0.011
Area (northern vs southern)	−0.006	−0.099 to 0.087	0.899
Sample size	−0.00006	−0.0002 to 0.00003	0.185
Economic level (developed vs not developed)	−0.082	−0.173 to 0.008	0.072
Average age	0.010	0.002 to 0.018	0.022
Quality score	−0.013	−0.051 to 0.024	0.482

## Discussion

In this systematic review, we estimated that in Chinese mainland, (a) the pooled prevalence rate of osteoporosis in diabetic patients is 37.8%, (b) old age and being of the female sex are both factors which correlate with a higher prevalence of osteoporosis and (c) economic level has a potential impact on the prevalence of osteoporosis in T2DM patients. To our knowledge, we are the first to systematically collect and analyze the studies utilizing DXA in T2DM patients.

According to the current results, the merged prevalence rate of osteoporosis in T2DM patients was much higher than the prevalence rate of primary osteoporosis in the Chinese Mainland reported by Chen (62). Notably, Chen has suggested that long-term high blood glucose might increase the risk for osteoporosis in diabetic patients largely. Additionally, some pathological mechanisms were related to this problem, such as deficiency or disorder of insulin, obesity, sexual hormone disturbance, and diabetic complications (63). In the early stage, Sosa and Wakasugi supposed that diabetes would not significantly impair bone metabolic status (64, 65). Recently, Athulya et al. also reported a negative difference in BMD between diabetic and non-diabetic subjects in India (66). Nevertheless, they noticed that the prevalence of osteoporosis was higher in the T2DM group than that in the control group.

Based on the results of the regression analysis, gender was a significant risk factor of osteoporosis in T2DM patients. We observed that the prevalence rate of osteoporosis in female T2DM patients (44.8%) was much higher than that in males (37.0%), and that they were higher than the results of primary osteoporosis reported by Chen respectively (62). Furthermore, age was another significant risk factor of osteoporosis in T2DM patients. The prevalence rate of osteoporosis (40.1%) in patients with an average age above 60 was higher than that in patients under 60 (26.5%). This result was consistent with the generally accepted concept that human bone mass would decline with age after reaching a peak.

In the subgroup analysis of the region, we did not detect an apparent difference between the prevalence rates of osteoporosis in T2DM patients residing in the southern and northern regions of China (37.6%, 38.2%). However, these two rates were higher than that of primary osteoporosis in the south and north China in Chen’s study (south: 23.17%, north: 20.13%) (62). Climates and eating habits, which were regarded as the influential factors of primary osteoporosis, appeared to have little impact on the prevalence rate of osteoporosis in T2DM patients in the south and north of China (67–70). The subgroup analysis of the economic level manifested that the prevalence rate of osteoporosis (32.7%) in diabetic patients from developed areas was significantly lower than that in patients from less developed regions (41%). Due to the in-equal distribution of medical resources, diabetic patients from developed areas are prone to receive better medical care and afford long-term medical expenses, which overall contributes to a more stable condition of diabetes.

On the contrary, a shortage of medical resources and a lower income level may aggravate the condition of diabetes, causing a high prevalence rate of osteoporosis. Notably, economic growth may be one of the primary reasons for the decline in osteoporosis prevalence in T2DM patients after the 2010 period (35.6%) compared with that in patients from 2001 to 2009 (42.3%). Therefore, we inferred that economic development was beneficial in establishing a stable T2DM condition, which is also crucial in preventing diabetic complications such as osteoporosis (71).

### Study Limitations

There were some limitations to this review. Firstly, we included 54 studies, but the number of involved participants still proves to be insufficient. Secondly, all patient information was obtained from hospitals and more women than men were involved in this study, which possibly leads to a higher pooled prevalence rate. Thirdly, a majority of included studies lacked the diabetes course information. Consequently, we were unable to analyze the associations between the diabetes course and the prevalence of osteoporosis in the subgroup analyses.

## Conclusion

This review demonstrates that osteoporosis is common in T2DM patients. Old age and being of the female sex proved to reflect a higher osteoporosis prevalence. Notably, economic development may favorably decrease the prevalence of osteoporosis in diabetic patients. However, it is necessary to conduct further studies so as to incorporate previous results, into clinical prevention for osteoporosis in T2DM patients.

## Ethical considerations

Ethical issues (Including plagiarism, informed consent, misconduct, data fabrication and/or falsification, double publication and/or submission, redundancy, etc.) have been completely observed by the authors.
